# Micro-Electromechanical Affinity Sensor for the Monitoring of Glucose in Bioprocess Media

**DOI:** 10.3390/ijms18061235

**Published:** 2017-06-08

**Authors:** Lorenz Theuer, Micha Lehmann, Stefan Junne, Peter Neubauer, Mario Birkholz

**Affiliations:** 1 Chair of Bioprocess Engineering, Department of Biotechnology, Technical University Berlin, ACK24, Ackerstr. 76, 13355 Berlin, Germany; lorenz.theuer@ri.se (L.Y.); micha.lehmann@hotmail.de (M.L.); stefan.junne@tu-berlin.de (S.J.); 2 IHP, Im Technologiepark 25, 15236 Frankfurt (Oder), Germany; birkholz@ihp-microelectronics.com

**Keywords:** bioprocess, affinity assay, viscometer, glucose monitoring, concanavalin A, bacterial culture media

## Abstract

An affinity-viscometry-based micro-sensor probe for continuous glucose monitoring was investigated with respect to its suitability for bioprocesses. The sensor operates with glucose and dextran competing as binding partner for concanavalin A, while the viscosity of the assay scales with glucose concentration. Changes in viscosity are determined with a micro-electromechanical system (MEMS) in the measurement cavity of the sensor probe. The study aimed to elucidate the interactions between the assay and a typical phosphate buffered bacterial cultivation medium. It turned out that contact with the medium resulted in a significant long-lasting drift of the assay’s viscosity at zero glucose concentration. Adding glucose to the medium lowers the drift by a factor of eight. The *c_glc_* values measured off-line with the glucose sensor for monitoring of a bacterial cultivation were similar to the measurements with an enzymatic assay with a difference of less than ±0.15 g·L^−1^. We propose that lectin agglomeration, the electro-viscous effect, and constitutional changes of concanavalin A due to exchanges of the incorporated metal ions may account for the observed viscosity increase. The study has demonstrated the potential of the MEMS sensor to determine sensitive viscosity changes within very small sample volumes, which could be of interest for various biotechnological applications.

## 1. Introduction

The continuous monitoring of specific metabolites and proteins is a key method towards establishing a thorough understanding and better control of bioprocesses in biotechnology. Continuously determined process data have the potential to improve the efficiency of methods and strategies like Design of Experiments (DoE), Quality by Design (QbD), and soft sensors. Continuous parallel and miniaturised sensors are especially of interest when miniaturised approaches are applied for bioprocess development, for example, when modern model-based DoE approaches with on-line refitting of model parameters are applied [1].

Regarding the substrates, glucose is of main interest, as it is the most commonly used carbon source in bioprocesses. Continuously operating enzyme-based glucose sensors have been reported since the 1980s [2] as has their use for the monitoring of the glucose concentrations in bioprocesses [3,4]. While the in-line use of biosensors in bioreactors is limited due to the need to resist the autoclavation process, other problems like enzyme stability, interfering and inhibiting substances in the medium [5], heat sensitivity of receptors [6], pH dependency of the enzyme activity [7], and sensor drifts have not been solved yet in ways that have led to sufficiently functional devices [8]. To date, only laboratory equipment and at-line systems (e.g., BioSenz Analyzer from Applikon, Delft, Netherlands) are used, and despite numerous publications and approaches, ready-to-use in-line biosensors for bioreactors are still unavailable.

Next to enzymatically operating biosensors, affinity-sensors for the continuous monitoring of biomolecules have gained attention in recent years. Similar to immuno-assays, an affinity-assay recognizes the analyte via specific binding to a receptor. However, as the interactions are non-covalent, non-catalytic, and reversible in nature, affinity-based sensors may be more robust and more easily applicable for continuous monitoring in bioprocesses compared to enzyme-based sensors. Binding constants of the analyte-receptor couple are normally in the range of 10^2^ to 10^5^ M^−1^. For comparison, binding constants of antibody-antigen couples are on the order of 10^8^ M^−1^ and below.

One example for such an affinity system that has been studied thoroughly is a sensor for glucose based on the plant lectin concanavalin A (ConA) acting as the receptor. ConA, with a size of 237 amino acids and a molecular weight of 26.7 kDa, utilizes two divalent metal ions for carbohydrate binding, which are located close to the ligand binding pocket. Cation binding sites S1 and S2 are occupied by Mn^2+^ and Ca^2+^ in the protein’s active state [9], although other divalent transition metal ions like Zn^2+^, Co^2+^, Ni^2+^, Cd^2+^, etc. may equally reside on S1 [10,11]. The incorporation of Mn^2+^ and Ca^2+^ results in a binding constant of 320 M^−1^ for ConA’s target molecules, which are α-d-glucosyl and α-d-mannosyl residues [12].

For glucose detection, the competitive binding between glucose and dextran to ConA is utilized. Dextran molecules are of globular shape and consist of α-1,3-linked glucose chains, at which ConA can bind with a binding constant of 1.5 × 10^4^ M^−1^ [12]. Different approaches were realized to measure the glucose concentration *c_glc_* in this competitive binding reaction [13]. Initial approaches made use of fluorescence detection [14,15] as did many of the following [16,17,18,19], but optical [20,21], osmotic pressure [22], and dielectric methods [23] were also investigated.

Reliable results were also obtained by transforming the *c_glc_* signal into a fluid-mechanical parameter, the dynamic viscosity *η* of the ConA/dextran assay [24,25]. This approach was first proposed and investigated by Ballerstädt and Ehwald [13,26]. Based on the tetrameric structure of ConA at physiological pH and the terminal glucosyl residues of a dextran molecule, mixtures of both become highly viscous. This viscosity depends on the mixing ratio and the number of bonds between ConA and dextran. Free glucose weakens the network, as glucose competes for carbohydrate binding sites, and, as a result, the viscosity decreases (see Table 1). To exploit the effect, the assay has to be separated from the analyte solution by a semipermeable membrane, which blocks the macromolecules [27,28,29]. In this way, the viscosity *η* of the assay becomes a function of the glucose concentration *c_glc_* outside the membrane.

Using modern microelectronic methods, a sensor chip for the measurement of viscosity was developed which utilizes a micro-electromechanical system (MEMS) [30]. The measurement of viscosity is carried out by using a quasi-electrostatic principle. For this purpose, a cantilever beam is moved by electric forces through the assay solution, with its velocity depending on the viscosity of the latter [31]. The sensor system was originally developed as a medical implant [32], i.e., for continuous glucose monitoring in human patients suffering from diabetes mellitus and has a very small size [33]. Various studies were performed on the affinity assay in contact with mammalian [34] and human tissue [35,36]. However, so far this approach has not been applied yet for glucose detection in bioprocess media.

In this work, we investigated if the sensor can be readily used in a typically applied defined mineral salt medium for the cultivation of *Escherichia coli*. Unexpectedly, and differing from the application in tissue environments, different medium components affect the sensor output and must be considered for application of the sensor.

## 2. Results and Discussion

The functionality of the sensor probe was first tested by inserting it into standard electrolyte (SEL) at *c_glc_* = 0 mM, and the switching time *t_sw_* was recorded. The parameter *t_sw_* of the MEMS served as a measure for the viscosity of the solution under test. It stands for the time it takes to bend the metallic cantilever beam to a defined position, where the bending is caused by electrical forces between the cantilever and ground plate (for construction details, see the Materials and Methods section). Typical bending amplitudes amount to about 1 µm, since the distance between the cantilever and ground plate initially is 2.5 µm, while *t_sw_* values were found to range between 5 and 300 µm in previous investigations [29,31], depending on the viscosity of test solutions. Next to viscosity, the switching time is also affected by the temperature and the electrical conductivity of the test solution.

**Figure 1:**
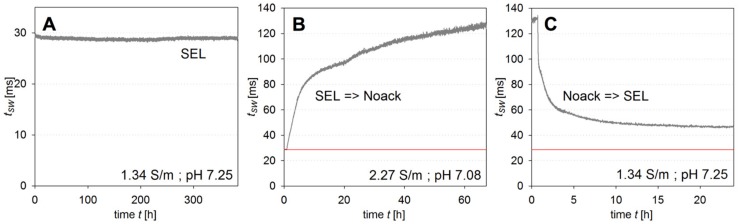
(**A**) Switching time *t_sw_* of the sensor probe in standard electrolyte (SEL) over 16 days. (**B**) Measurement in Noack medium exhibiting with drifting *t_sw_* values (**C**). Subsequent measurement in SEL brought *t_sw_* values almost back to its starting point, the red line marks the mean value of the 16-day measurement shown in (**A**).

During the test measurement *t_sw_* was recorded every one minute for 16 days. It can be seen from Figure 1A that *t_sw_* stayed constant at a mean value of 28.9 ms with a standard deviation of 0.2 ms, i.e., with variations of less than 1%. The data demonstrate a remarkable stability of the assay and the sensor chip throughout more than 2.3 × 10^4^ measurements. In particular, the stable sensor data indicates the semipermeable membrane between the sensoric assay and the test solution to be free of defects, which would cause leaking out of ConA and/or dextran and a drift of sensor signals.

Following this first functionality test, the sensor was placed into a typical bacterial phosphate buffered mineral salt medium [37] with *c_glc_* = 0 mM, abbreviated by MS or Noack in the following. Figure 1B shows the course of *t_sw_* for a time span of 3 days. The switching time *t_sw_* increases rapidly, exhibiting rates between 1 and 10 ms h^−1^ without reaching a stable end value within the duration of the experiment.

Returning the sensor to SEL with *c_glc_* = 0 mM (Figure 1C) results in a fast decrease of the switching time *t_sw_* which then stabilizes at a value higher than the one prior to exposure to the mineral salt medium. This shows that the response consists of a reversible and an irreversible part.

We safely exclude the effect caused by erroneous measurements, because it could be reproduced and because of the high stability of sensor operation as demonstrated in SEL. It has been shown by [31] that glucose concentrations *c_glc_* can be derived with 1% precision from the switching time *t_sw_* in SEL medium.

An important physical parameter is the electrical conductivity σ, which naturally depends on the concentrations of electrolytes and their mobility in the solution. The conductivity roughly doubles when going from SEL to Noack medium. An electrical field is operative between the MEMS cantilever and its bottom plate due to the quasi-electrostatic deflection scheme. The energy of the high-frequency (HF) field is also transformed into kinetic energy of the surrounding ions and the share of the latter increases with the ion concentration. It thus takes an increased amount of time for the cantilever to reach the switch-off position if the conductivity σ is higher (d*t_sw_*/dσ > 0).

In principle, the change of *t_sw_* is in accordance with the rise of conductivity. The conductivity difference, however, cannot explain the time constant of hours and days, on which *t_sw_* steadily increases. Diffusion constants *D* in the order of 1 to 2 × 10^−9^ m^2^ s^−1^ for electrolytes like Na^+^ and Cl^−^ would predict electrolyte concentrations *c_el_* to equilibrate within seconds in the cavity and the test solution after changing the medium.

Another difference between SEL and Noack medium is the pH value. It drops from 6.85 to 6.65. Although this could principally result in a certain disaggregation of ConA tetramers, this disaggregation should lead to a weakening of the ConA/dextran network and therefore to a drop of viscosity and *t_sw_*. In contrast, the viscosity remained nearly constant (data not shown).

In fact, the large time constants suggest biochemical rather than physical causes account for the observed *t_sw_* increase. A large part of the sensor response is reversible, and the speed in which the switching time drops after returning the sensor to SEL implies a mechanism that can be reversed simply by diffusion of Noack medium components out of the sensor cavity. The irreversible change of viscosity, on the other hand, seems to not be dependent on the concentration of the influencing medium components.

In order to investigate which component might be responsible for the change of viscosity, different components of the medium were separately added to SEL in the same concentration as they are present in Noack medium. The tests were performed at *c_glc_* = 0 for MgCl_2_, NH_4_Cl, FeCl_3_, and the phosphate buffer components. Each solution was tested by monitoring ∆*t_sw_* with the sensor probe, starting from the value initially measured in SEL. After monitoring for 24 h, the sensor was returned to unmodified SEL solution until no further change in *t_sw_* could be observed.

**Figure 2:**
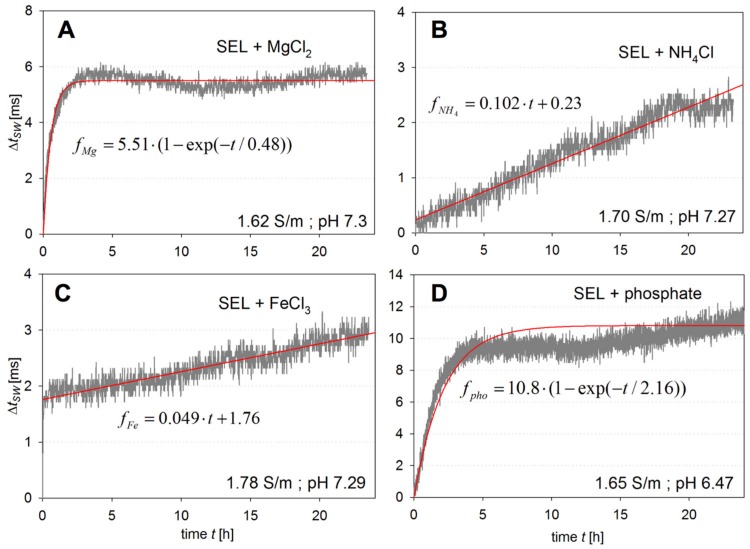
Difference in switching time ∆*t_sw_*(*t*) during the observation period measured in SEL including the individual components of mineral salt (MS) medium: (**A**) MgCl_2_; (**B**) NH_4_Cl; (**C**) FeCl_3_; (**D**) phosphate buffer components.

Figure 2A–D shows that all the components cause an increase in *t_sw_*. The strongest increase in *t_sw_* of around 9 ms in 5 h was caused by the addition of phosphate buffer (Figure 2D). For the addition of FeCl_3_, the effect of an increase in conductivity, as discussed above, is clearly obvious (Figure 2C). The short response time is caused by the fast diffusion of Fe^3+^ and Cl^−^ into the sensor cavity. Another fast increase in *t_sw_* occurs with MgCl_2_. However, a longer time constant of about 1 h can also be observed, but the increase stops after approximately 2 h. On the other hand, the presence of NH_4_Cl and FeCl_3_ leads to a constant increase of *t_sw_* during the observation time. Again, the initial increase can be understood by the higher conductivity of the solutions. The later increase has to have other reasons than the diffusion of media components into the sensor cavity, which should be at equilibrium at that point of time. In fact, the persistent *t_sw_* increase suggests an induced interaction of assay components among themselves.

**Figure 3:**
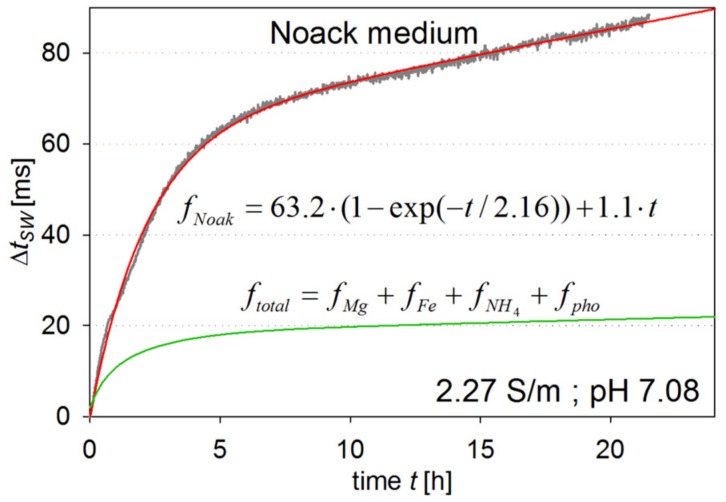
Comparison of ∆*t_sw_* transients after transferring the sensor probe from SEL to MS medium (**top**) and the sum of individual components of medium as derived from their fitting functions (**bottom**).

Fit functions *f* of the measured transient curves *f*_Mg_, *f*_Fe_, *f*_NH4_, and *f_pho_* are also displayed exhibiting either linear or exponential behaviour. A summation of all four fit functions and a comparison of the ∆*t_sw_* response to the one in Noack medium are shown in Figure 3. The comparison shows that the component effects do not follow a linear superposition, but are reinforcing each other. As for possible causes, we suggest the following: (i) An exchange of the two divalent metal ions for carbohydrate binding within the lectin could lead to a decreased binding constant which would strengthen the ConA/dextran network. One or more chemical intramolecular binding reactions between (ii) dextran or (iii) ConA molecules could also strengthen the network of the assay components; (iv) The electro-viscous effect caused by a bigger protein ion-cloud. At the actual point of investigation, other possible explanations cannot be excluded, but the mechanisms mentioned appear the most likely ones based on our experimental findings.

(i) When the two cation binding sites S1 and S2 are occupied by ions, the protein undergoes a conformational change that involves the isomerization of a non-proline peptide bond from *trans*- to its *cis*-conformation. The isomerization presents a certain energy barrier that needs to be overcome. This change has been called “locking” and it largely increases ConA’s affinity to the bound ions. It has been shown that only locked ConA can bind to carbohydrates, but also metal-free ConA can be in its locked/native state. Incubating with excess carbohydrates results in a shift of unlocked/locked equilibrium of metal free ConA from around 12% to over 60% of the locked conformation in an experimental set up. Varying the ion species in S1 and S2 has shown little change in binding strength to the ligand. However, the most important divalent metal cation Ca^2+^ is missing in the growth medium, and even though no direct explanations can be found in the literature, it is evident that ConA is a very cation sensitive protein with three cation binding sites, and therefore variations in ligand binding strength due to changes in specific cation concentrations cannot be ruled out.
(ii) We also considered the phosphorylation of glucosyl residues that could lead to covalent bonds between dextran molecules, which would lead to a permanent increase in viscosity by strengthening the molecular network of the assays. In the lab, phosphorylation of dextran was achieved under exclusion of water at high temperatures (90 and 120 °C, respectively for 6 h, only approximately 10%) and low pH [38,39]. Therefore, phosphorylation appears rather unlikely to happen spontaneously under the conditions present in the sensor cavity.
(iii) Some of the salts used in Noack medium are applied in protein crystallization protocols, namely NH_4_^+^, SO_4_^+^, and phosphate. Although their concentration in the medium is much smaller, the ions mentioned belong to the most effective salting-out agents of the Hofmeister series [40]. Thus, it might be expected that aggregation of ConA to higher mers than tetramers strengthens the intermolecular network between assay components and causes a higher viscosity. This assumption is supported by the fact that the applied concentration of ConA is close to its solubility limit in solutions of electrolytes used in the medium [41].
(iv) Agglomeration induced by electrolytes and the associated increase in viscosity were already described by Kim and Myerson for lysozyme [42]. Concentrations of the involved ions and the apparent time constants were of the same order of magnitude as observed in this work. Their study also pointed to the electro-viscous effect that accounts for the property of proteins to surround themselves with a cloud of dissolved ions. The cloud composition, which depends on ionic species and concentrations, determines the forces necessary to move the protein molecule within the solvent, and thus the viscosity of the solution.

It is evident from the considerations given above that the intention to use the affinity-viscometric sensor probe for continuous glucose monitoring in bioprocesses faces several challenges. It has to be emphasized, however, that these objections do not apply to the usage of the sensor probe in human body implants, where variations in δ*T* and δσ, for example, can be neglected and where pH is effectively buffered by bicarbonate.

**Figure 4:**
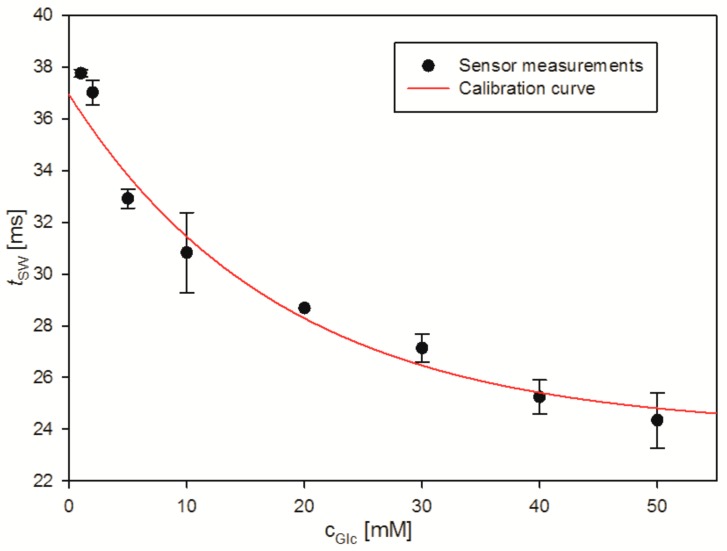
Calibration curve measured before the cultivation monitoring experiment in Noack medium with defined glucose concentrations between 1 and 50 mM during 6.5 h.

In a next step, a new probe was used to monitor changes of *c_gls_* throughout a cultivation experiment. As a preparation, the sensor was calibrated in MS medium with glucose concentrations ranging from 1 to 50 mM, the results of which are shown in Figure 4. During the course of the calibration measurements, *c_glc_* = 1 mM was measured twice after 0.5 and 3 h, showing a mean *t_sw_* value of only 3.53 ms with a standard deviation increasing from 0.15 to 0.3 ms. Compared to the sensor response in Figure 1B, this results in only around 12% of the drift observed in Noack medium without glucose. Having glucose in the sensor cavity therefore decreased the sensor response to the MS medium by a factor of eight.

**Figure 5:**
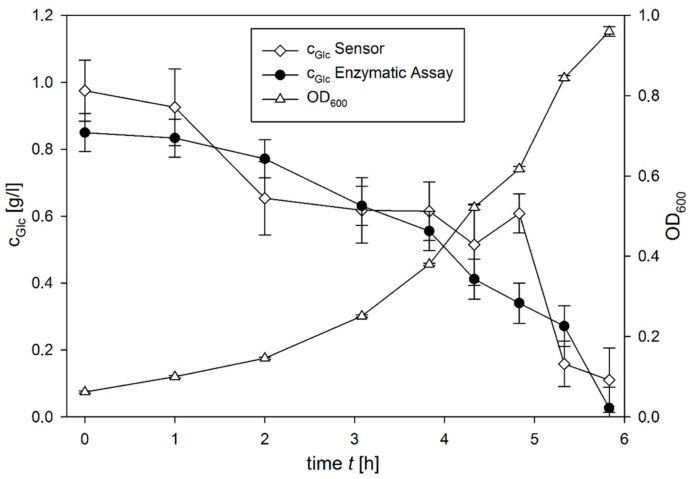
Growth curve of a BL 21 *Escherichia coli* cultivation in Noack medium. The cell growth was monitored by optical density at 600 nm (OD_600_). For glucose measurements, samples were removed from the cultivation and analysed with the glucose sensor and a commercial enzymatic glucose hexokinase assay.

In order to monitor a real cultivation, nine samples were taken from the cultivation medium for off-line measurements. The increase in turbidity measured as absorbance at a wavelength of 600 nm (OD_600_) confirms bacterial growth throughout the cultivation. Meanwhile a steady decrease of glucose concentration in cultivation supernatant could be shown both by the sensor and reference measurement with an enzymatic assay (see Figure 5). It is important to note that during the monitoring experiment the sensor was submerged in the cultivation samples for intervals between 20 and 30 min.

The *c_glc_* values measured with the glucose sensor were similar to the measurements with the enzymatic assay, with a difference of less than ±0.15 g·L^−1^ for every measurement, with the exception of an outlier at 5 h. This shows that in a suitable application, the sensor produces both a reliable calibration curve and measuring results that are similar to an established method. The relative constancy of the glucose concentration, and thus the larger error in the glucose concentration, between 2 and 5 h of the cultivation is possibly related to the continuous pH decrease, which results in a lower viscosity and thus smaller *t_sw_* values, most probably due to a loss of the intramolecular interactions in the ConA tetramer. This pH dependency is critical for the application of the sensor, which thus would have its application potential mainly for pH controlled cultivations which are standard in industrial bioprocesses.

The difference in viscosity response to medium containing either glucose or no glucose points towards (a) agglomeration of ConA and (b) the change of the binding constant by an exchange of the two divalent metal ions for carbohydrate binding within the lectin. Both mechanisms would be affected by the presence of glucose. Statistically, glucose binds about 40 times stronger to ConA than glucosyl residues, so any strengthening of the ConA binding would not result in as much of a rise in viscosity.

While the viscosity-based MEMS sensor for glucose presented here seems to be promising, this study shows that, for applications which apply continuously changing media, special optimization of the viscous polymer network is necessary. However the presented sensor is directly applicable under conditions where a defined assay environment is created, for example, by the fast at-line analysis of glucose in samples collected from parallel cultivations in a liquid handling station in experimental approaches [1].

## 3. Materials and Methods

The preparation of sensor chips on CZ-Si 200 mm wafers was conducted by semiconductor technology using the IHP-proprietary 0.25 µm SGB25V technology [43]. The MEMS cantilever made from bio-stable TiN was initially embedded with interlayer dielectric layers [44] from SiO_2_, but was released in a chemical etching step after bringing the wafer out of the clean room. For this purpose, the liquid from the last rinsing procedure was superseded with CO_2_ by critical point drying in order to prevent unwanted static friction or stiction of the cantilever to the bottom plate [45]. Afterwards, the bond pads for the electrical connection were provided with Au stud bumps and the sensor chips were separated using stealth dicing [46]. Every chip was then flip-chip bonded to a flexible circuit board for electrical connection.

**Figure 6:**
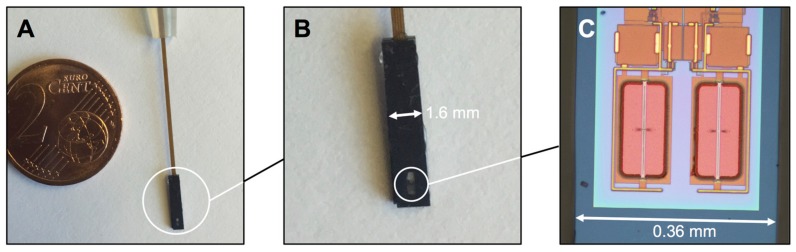
(**A**) Sensor probe (white circle); (**B**) Magnification of the sensor probe showing the cooling body, cover plate and semipermeable membrane; (**C**) Optical microscope picture of the micro-electromechanical system (MEMS) part of the sensor chip prior to integrating the semipermeable membrane and filling with the affinity assay. The measurement MEMS is given on the left and the reference MEMS is provided on the right-hand side of the figure. Golden-coloured cantilevers can be seen to stretch over the ground plates (orange-brown) in both cases. Due to the architecture of the semiconductor layer stack, the cantilevers have a height of 2.5 µm above the ground plate, such that the configuration of both forms a kind of parallel plate capacitor.

Bonded chips were glued into a cooling body in order to dissipate the thermal energy generated during measurements. Integrating a semipermeable membrane with a cut-off at 2.2 nm [29] into the cooling body forms a cavity with a volume of about 1 µL (see Figure 6A,B). The cavity was filled with the affinity assay through a channel at the back, which was sealed afterwards. The affinity assay consisted of ConA and dextran dissolved in SEL solution added with functional cations [47] and Tris buffer.

The measured quantity of the sensor MEMS is the switching time *t_sw_*, which is the time needed to deflect the cantilever to a defined position. The sensor chips are subjected to a direct current (DC) voltage *V_dd_* of 3.3 V, which is transformed into a high-frequency alternating current (AC) voltage of 3.2 GHz by a ring oscillator circuit that is applied to the cantilever [31]. To compensate for production variations, a control voltage *V_ctrl_* is added that enables chip-specific adjustments of switching time *t_sw_*. Variations of environmental conditions are compensated by using two MEMS cantilevers: one for the measurement and a second one with a rigid, unbendable beam acting as reference.

In an extensive study of the sensor chips, X-formed cantilevers were investigated, but also Y- and I-shaped geometries can be prepared [33]. In this work, I-shaped cantilevers with a width of 6 µm and a length of 150 µm were used (Figure 6C). Switching time *t_sw_* and glucose concentration *c_glc_* are related by the characteristic function

(1) *t_sw_* = *k*_1_ exp(−*c_glc_*/*k*_2_) + *k*_3_

with coefficients *k_i_* to be determined for each sensor probe in a calibration step prior to usage. The viscosity strongly depends on temperature *T* of the measured liquid. Therefore, a band gap circuit for *T* measurement was integrated into the sensor chip [48] in order to allow for a *T*-dependent estimation of *k_i_*.

Measurements were made by placing the sensor probe in a 1.5 mL tube containing the sample. The tube was placed inside a thermostat (Labnet Accublock Mini Digital Dry Bath, Edison, NJ, USA) to keep the temperature at 37 °C. The flex cable was connected to the system board that houses a microcontroller (Texas Instruments MSP430, Dallas, TX, USA) and a radio module. The system board received its power from an Agilent E360A DC power supply, Santa Clara, CA, USA.

Conductivity measurements were made at 37 °C with a Mettler-Toledo FiveEasy FE30, Gießen, Germany. Measurements of pH were performed at room temperature with a Schott pH meter CG824, Mitterteich, Germany. Standard electrolyte (SEL) solution, the affinity assay, and mineral salt medium [37] were prepared according to Table 1. Deionized water (ρ ≥ 18 MΩ·cm) was used throughout all experiments.

The cultivation for a monitoring experiment was performed in a 250 mL magnetically stirred double wall glass bioreactor (Ochs Laborfachhandel, Bovenden, Germany) with a BL 21 *E. coli* strain. A starting OD_600_ of 0.1 and an initial glucose concentration of 1 g·L^−1^ in mineral salt medium (in accordance with [37]) were used. Medium temperature was adjusted to 30 °C. Samples were taken hourly. OD_600_ was measured photometrically with an Ultraspec 3300 Pro from Amersham Biosciences, Freiburg, Germany. After centrifugation of the samples at 10^5^ g for 5 min, the supernatant was filtered and conductivity, pH, and *c_glc_* were measured. The latter was determined with the MEMS sensor and referenced to the Glucose Hexokinase FS kit (DiaSys Diagnostic Systems, Holzheim, Germany).

## 4. Conclusions

In this study, a sensor intended for continuous glucose monitoring and developed for medical applications was tested in a bioprocess environment. Its functionality relies on a MEMS chip and a biochemical affinity assay containing ConA and dextran. A mineral salt culture medium for *E. coli* bioprocesses was chosen as a representative model system. The study combines an investigation focused on the interactions of the affinity assay with the components of the medium and an off-line monitoring experiment of an *E. coli* cultivation. Whereas an unprecedented stability of sensor operation could be observed in standard electrolyte, the measurement signal *t_sw_* experienced a significant drift after bringing it in contact with bioprocess medium in the absence of glucose. Interestingly, the addition of glucose resulted in an eight times lower response. The effect may be caused by the aggregation of ConA, the cross-linking of dextran molecules, the change of binding constants due to exchange of metal ions in ConA, or the electro-viscous effect, which would modulate the viscosity independently of analyte concentration.

While the sensor may be directly applied off-line with diluted samples at higher glucose concentrations, the application of the sensor as an on-line tool for glucose monitoring in bioprocess media appears challenging at the current state of development. While the pH dependency of the signal is important to consider, it is not a problem, as most biotechnological processes are performed at constant pH, although the shift of *t_sw_* in a phosphate buffered medium has to be considered in the calculation of glucose concentrations. Also, changes in divalent ions over the run of the cultivation may eventually influence the signal. Thus, further developments may have to consider a second reference chamber with a membrane which cannot be penetrated by glucose.

The presented measurements of the new sensor MEMS have also demonstrated its ability to determine viscosity within very small sample volumes in the pL range. This should be of interest for, e.g., the investigation of aggregation in the early stages of protein crystallization and other applications in bio-rheology or biotechnology in general.

## Figures and Tables

**Table 1:** Composition of media used. SEL, standard electrolyte.

Standard Electrolyte SEL			Mineral Salt Medium [37]		
Component	g·L^−1^	mM	Component	g·L^−1^	mM
Tris/HCl	1.21	7.7	KH_2_PO_4_	2.72	20
NaCl	7.76	133	Na_2_HPO_4_ × 2H_2_O	3.56	20
MnSO_4_ × H_2_O	0.169	1.00	NaCl	5.00	85.6
CaCl_2_	0.147	1.32	Na_2_SO_4_	1.10	7.74
NaN_3_	0.5	7.7	NH_4_Cl	0.50	9.35
			MgCl_2_ × 6H_2_O	0.04	0.197
**Affinity assay = SEL + ConA + dextran**			FeCl_3_ × 6H_2_O	0.008	0.030
Dextran T2000	70	0.035	MnSO_4_ × H_2_O	0.004	0.024
ConA	7	0.264	Thiamine	0.05	0.148

## Abbreviations

ConA	Concanavalin A
MEMS	Microelectromechanical system
OD_600_	Optical density at 600 nm
